# Prevalence of Childhood Stunting and Its Associated Factors in Bangladesh: Quantile Regression Modeling Approach

**DOI:** 10.1155/jnme/5517189

**Published:** 2026-10-01

**Authors:** Israt Jahan, Md. Israt Rayhan, Kanis Fatama Ferdushi, Mirza Nazmul Hasan, Nahid Sultana

**Affiliations:** ^1^ Department of Statistics Shahjalal University of Science and Technology Sylhet Bangladesh sust.edu; ^2^ Institute of Statistical Research and Training (ISRT) University of Dhaka Dhaka Bangladesh du.ac.bd; ^3^ University of Sheffield Sheffield UK sheffield.ac.uk

**Keywords:** height-for-age Z-score, Kruskal–Wallis test, quantile regression, stunting, under-5 children

## Abstract

**Background:**

Stunting is a key public health concern among under‐five children in Bangladesh. It affects approximately one‐quarter of children under 5 years of age, which is higher than global averages and insufficient to meet the goal of the World Health Organization (WHO) for reducing stunting to 15%. So, further research is needed to better understand the current dynamics of stunting and to develop pragmatic policies based on the most recent nationwide data which will provide valuable insights into the factors influencing stunting in Bangladesh. This study investigates the prevalence and risk factors of stunting among children under five using the most recent national data.

**Methods:**

Data were analyzed from the Bangladesh Demographic and Health Survey (BDHS) 2022. The data collection for the 2022 BDHS took place from June 27, 2022, to December 12, 2022. This survey used stratified sampling and selection is made in two stages. In the first stage, 675 enumeration areas (EAs) were picked from each stratum and a fixed number of 45 households per EA were selected in the second stage. The final data contain 8784 children under 5 years. After cleaning the flagged cases and missing values of related variable, the dataset comprises information on weighted sample of 3664 children. In bivariate analysis, a nonparametric Kruskal–Wallis test was employed for violating the parametric assumptions. Subsequently, robust quantile regression models were utilized to evaluate the impact of various independent variables on height‐for‐age Z‐score (HAZ) across different quantiles.

**Results:**

Results reveal that the prevalence of stunting was 23%, with 17.8% and 5.2% classified as moderately and severely stunted, respectively. The quantile regression analysis shows significant heterogeneity in the determinants of child growth across the HAZ distribution. Predictors with stronger effects among moderately and severely stunted children (lowest HAZ quantile) include older child age, high birth order, twin births, low maternal BMI, low maternal education, lack of media exposure, low paternal education, and poor household wealth. Older child age was consistently associated with lower HAZ across all quantiles. The positive effects of maternal education, maternal overweight status, media exposure, and household wealth index are strongest at the lower HAZ quantiles. Children with higher birth orders have lower HAZ through all quantiles compared to first‐born children. Twin births are associated with lower HAZ in the lower and middle quantiles. Several predictors, particularly maternal education, maternal nutritional status, and household wealth, exhibit higher prognostic value for severe and moderate stunting than for children in higher HAZ quantiles.

**Conclusions:**

Stunting remains a significant public health concern among children in Bangladesh. The effects of child, maternal, household, and health‐related factors varied across the HAZ distribution, with several predictors showing stronger associations among children with lower HAZs. Quantile regression provided important insights beyond conventional analyses by identifying factors with stronger effects among children in the lower tail of the HAZ distribution.

## 1. Introduction

Child undernutrition means deficiencies in a child’s intake of energy and essential nutrients [1]. Poor nutrition impairs both physical and mental development during childhood [2]. Stunting is a key indicator of inadequate child development, which is measured by a low height‐for‐age Z‐score (HAZ) [3]. HAZ is a standardized anthropometric index which is used to measure a child’s linear growth relative to an international healthy reference population [4], and a low HAZ is the result of long‐term deficiencies. According to the World Health Organization (WHO), HAZ cut‐points of < −2 and < −3 are recommended for children under 5 years for classifying moderately and severely stunted, respectively [5]. Stunting reflects chronic undernutrition and hinders the achievement of full linear growth potential [6]. Although stunting is commonly used as an indicator of chronic undernutrition, short stature is not exclusively caused by inadequate dietary intake. Child linear growth is influenced by a complex interaction of nutritional, biological, environmental, and socioeconomic factors, including maternal health, intrauterine growth restriction, recurrent infections, environmental enteric dysfunction, inadequate water, sanitation and hygiene conditions, and genetic influences [7–9]. Consequently, improvements in nutrition alone may not always translate into proportional gains in child linear growth.

Stunting is a vital contributor to morbidity as well as mortality among under‐five children [10]. The long‐term consequences of childhood stunting are thoughtful [11], because it often harms cognitive abilities. It also hinders optimal development [12], leading to declining performance levels. That is, stunting has far‐reaching effects on society as it reduces human capital, which is closely linked to poverty and insufficient healthcare services [13]. Malnourished children are also more vulnerable to illnesses compared to their well‐nourished child [14]. Therefore, a proper diet is essential during childhood.

In 2020, about 149 million children under the age of five were stunted globally [1], with about 79 million of them living in Asia mainly in Southeast and South Asia, 1.8 million in Europe, 61.4 million in Africa, and 5.8 million in Latin America and the Caribbean [15]. The prevalence of stunting among Bangladeshi children under 5 years old remains concerning, with a rate of 24% in 2023 [16]. Although this represents a reduction from 42% in 2013 [16], the rate is still alarmingly high. Stunting rates are higher in rural areas across the country’s divisions [17] as well as millions of children in densely populated urban centers like Dhaka continue to suffer from undernutrition [18]. Both governmental and nongovernmental organizations have taken various initiatives to reduce the prevalence of stunting. But the current interventions are insufficient to meet the goal of WHO for reducing stunting to 15% [19]. Therefore, further research is needed to better understand the current dynamics of stunting and to develop pragmatic policies.

Numerous studies have been conducted to explore the prevalence and determinants of stunting in Bangladesh. Chowdhury et al. [20] used data from the Multiple Indicator Cluster Survey (MICS) 2018–19 to identify risk factors for childhood stunting through multiple logistic regression analysis. The study found that living in the Sylhet division, increasing child age, having several under‐five children in a household and using unhygienic toilets were significant risk factors for stunting. In contrast, higher parental education and a higher wealth index were protective factors. Similarly, Islam et al. [21] studied the data of BDHS 2017–18 and identified maternal abnormal body mass index (BMI), maternal age, child age, birth order, paternal education, wealth index, and sanitation facilities as key determinants of stunting by using binary logistic regression model. Rahman et al. [22] analyzed BDHS 2004 data and found that region, father’s education, toilet facilities, and wealth index were significantly associated with stunting by applying multivariate logistic regression models. Mansur et al. [23] also used the binary logistic regression model in BDHS 2004 data and showed that children with working mothers, those living in the Sylhet division, and in urban areas were at higher risk of stunting. In contrast, children from wealthier households and those with mothers who had higher BMIs were at lower risk. Das et al. [17] applied the ordinal logistic regression (OLR) model to identify the determinants of child malnutrition by using the data of BDHS 2004 and determine that age of child, birth interval, mothers’ education, maternal nutrition, household wealth status, child feeding index, and incidence of fever, ARI, and diarrhea were the significant predictors of child malnutrition.

Most of the previous studies in Bangladesh have examined stunting as a binary, ordinary outcome or have focused on average differences in HAZ using conventional regression models. But these models give the estimate of covariates based upon the mean of the dependent variable (HAZ). Also the estimates are influenced by outliers [24]. In addition, covariates are not homogeneous through the distribution of HAZ, and their influence can differ across the quantiles of HAZ. Furthermore, stunted children are at the lower tail of the distribution of HAZ, not in the middle. So, these models can fail to accurately identify the dependent variable’s distribution when the data do not satisfy the model assumptions. A major disadvantage of these regression models is to classify the dependent variable based on specific thresholds. It may lose statistical power in the estimation of odds ratio [25].

Currently, the quantile regression (QR) model, introduced by Koenker, is broadly applied in different fields for statistical research. The advantage of using the QR model is that it can estimate the effect of covariate at any percentile of the distribution even when the data do not meet the normality assumption and also robust to outlier [6]. WHO stunting categories are based on predefined HAZ cut‐off points, whereas QR examines associations across different points of the continuous HAZ distribution and does not classify children into stunting categories. QR is particularly useful in child growth research because it allows the examination of whether associations between independent variables and HAZs differ across the outcome distribution. Factors influencing children with relatively poor growth outcomes (lower HAZ quantiles) may not have the same magnitude of association among children with average or better growth outcomes. Therefore, QR can provide a more comprehensive understanding of growth inequalities than conventional mean‐based regression approaches. Abdulla et al. [26] applied the QR model to capture the heterogeneous effects of risk factors for stunting by using BDHS 2017–18 data. But no research has yet explored childhood stunting using the latest BDHS 2022 dataset. Therefore, this study utilizes the most recent nationwide data, which will provide valuable insights into the factors influencing stunting in Bangladesh.

The aim of this study is to examine the association of various child, parental, household, and health‐related factors on stunting among Bangladeshi children under 5 years old by applying a robust QR model at different points of the HAZ distribution.

## 2. Materials and Methods

### 2.1. Source of Data

The research relies on the secondary data which comes from the “Bangladesh Demographic and Health Survey (BDHS) 2022,” the ninth DHS survey conducted in Bangladesh. The survey was led through a collaboration between NIPORT, Mitra & Associates, and ICF International, with the primary goal of providing up‐to‐date information on maternal and child health, including nutritional status. The BDHS 2022 was carried out across 675 clusters, comprising 30,375 households [16]. The data consist of 8784 under‐five children and anthropometric data available for 4105 children. After cleaning the flagged cases and missing values of related variable, the final sample consists of 3647 children under the age of 5 years. Weighted sample of 3664 children are used for the final analysis to ensure the sample accurately mirrored the population of the country. An explanation of the sampling methodology and the mathematical adjustments used for the weighted sample can be found in the published 2022 BDHS report. Figure 1 illustrates the sample selection procedure.

**FIGURE 1:**
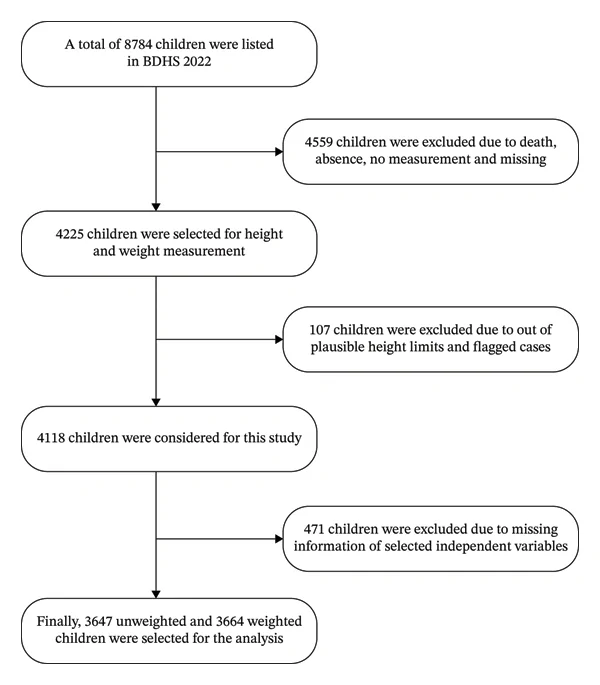
Flowchart of the sample selection procedure.

### 2.2. Variables

#### 2.2.1. Dependent Variable

The dependent variable in this study is the HAZ, which reflects linear growth retardation and cumulative growth deficits. According to the WHO child growth standards, HAZ cut‐offs of < −2 and < −3 are used to classify children under 5 years of age as moderately and severely stunted, respectively.

#### 2.2.2. Independent Variables

The independent variables for this study are selected based on the variables available in the BDHS 2022 dataset and insights from relevant reports. These variables encompass child, parental, household, and health‐related characteristics for analyzing the HAZ.

*Child-related characteristics* include age in months (≤ 6, 7–23, 24–47, 48–59), sex (male, female), birth order (1st, 2nd–3rd, 4th, or higher), and twin birth status (no, yes).

*Maternal characteristics* include current age (≤ 18, 19–24, 25–34, 35, or older), age at first birth (< 18, ≥ 18), BMI (underweight: < 18.5 kg/m^2^, normal: 18.5–24.9 kg/m^2^, overweight: ≥ 25 kg/m^2^), education level (illiterate, primary, secondary, higher), current working status (no, yes), and access to media (no, yes).

*Paternal characteristics* include education level (illiterate, primary, secondary, higher) and occupation (no work, nonagricultural work, agricultural work, service).

*Household and health-related characteristics* include region or division (Dhaka, Barisal, Chittagong, Mymensingh, Khulna, Rangpur, Rajshahi, Sylhet), place of residence (urban, rural), religion (Muslim, non‐Muslim), wealth index (poor, middle, rich), number of household members (≤ 4, 5–7, ≥ 8), number of children under five in the household (≤ 2, ≥ 3), household head’s sex (male, female), source of drinking water (improved, unimproved), type of toilet facility (improved, unimproved), recent diarrhea (no, yes), and fever in the last 2 weeks (no, yes).

### 2.3. Statistical Analysis

The assumptions for parametric tests are assessed using the Kolmogorov–Smirnov and Breusch–Pagan tests. Since these assumptions are violated, robust nonparametric methods are applied instead of parametric techniques. To examine the bivariate association between HAZs and the independent variables, the Kruskal–Wallis test is employed. Finally, QR models are used to evaluate the effect of each independent variable at different quantiles of HAZ because it does not require the normality assumption, is less sensitive to outliers, and provides robust estimates of the conditional distribution of the outcome variable across different quantiles. Stunting is defined using predefined HAZ cut‐off points (< −2 SD for stunting and < −3 SD for severe stunting) according to WHO Child Growth Standards. In contrast, QR models the continuous HAZ distribution and estimates associations at different quantiles of HAZ. Therefore, HAZ quantiles do not correspond to stunting categories; rather, they allow assessment of whether associations between independent variables and child growth differ across the distribution of HAZs. In this study, the WHO cut‐off‐based classification is used to estimate stunting prevalence and the QR approach is used to model continuous HAZs.

The QR model is defined as

(1) QτYi=α0τ+α1τXi1+⋯+αpτXip i=12,,…,n,

where *Q*_*τ*_(*Y*_*i*_) is the *τ*th quantile of the conditional distribution of *Y*_*i*_ given covariates *X*_*i*1_ … *X*_*i* *p*_, and *α*_0_, *α*_1_, …*α*_*p*_ are the regression parameters.

The influence of each independent variable is measured by fitting QR models in different quantiles (0.0547, 0.235, 0.5, 0.75, 0.95). The lower quantiles (*τ* = 0.0547 and *τ* = 0.235) were selected because they approximately corresponded to the cumulative proportions of children classified as severely stunted (5.2%) and stunted (23%) in the study population, allowing examination of associations among children located at the lower tail of the HAZ distribution. The remaining quantiles (*τ* = 0.50, 0.75, and 0.95) were included to assess associations across the median and upper portions of the HAZ distribution.

The parameters of the QR models are estimated by minimizing the weighted absolute sum of deviations [27].

All analyses were conducted in SPSS 25 and R 4.3.2.

## 3. Result

Table 1 provides a summary of the child, parental, household, and health‐related characteristics of the participants, along with 95% confidence intervals of the proportions. The findings reveal that 17.8% and 5.2% of the children are moderately and severely stunted, respectively. The results also show that male children make up just over half of the sample (51.5%), approximately 41.2% of the participants are from poor households, and about one‐quarter (24.6%) are from the Dhaka division. The majority (73.9%) of the participants reside in rural areas, and only 12.5% of household heads are women. Additionally, around 33.8% of mothers gave birth for the first time before the age of 18, and 64% of mothers are illiterate.

**TABLE 1:** Summary of selected background characteristics, BDHS 2022 (sample size = 3664 (weighted)).

Variables		Frequency (%)	95% C.I.
*Stunting status*			
Not stunted		2821 (77)	(75.1–77.8)
Moderately stunted		653 (17.8)	(16.9–19.4)
Severely stunted		190 (5.2)	(4.7–6.2)

*Child characteristics*			
Age (in months)	≤ 6	420 (11.4)	(10.4–12.4)
	7–23	1086 (29.6)	(28.4–31.3)
	24–47	1444 (39.4)	(37.8–40.9)
	48–59	714 (19.5)	(18.2–20.8)

Sex	Male	1888 (51.5)	(50.3–53.5)
	Female	1776 (48.5)	(46.5–49.7)

Birth order	1st	1307 (35.7)	(33.8–36.9)
	2nd‐3rd	1978 (54)	(52.3–55.4)
	4th or higher	379 (10.3)	(9.8–11.8)

Child is twin	No	3620 (98.8)	(98.4–99.1)
	Yes	45 (1.2)	(0.9–1.6)

*Mother’s characteristics*			
Current age (years)	≤ 18	182 (5)	(4.2–5.6)
	19–24	1273 (34.7)	(33.3–36.4)
	25–34	1778 (48.5)	(46.9–50.1)
	35 or more	433 (11.8)	(10.8–12.9)

Age at first birth	< 18	1240 (33.8)	(32–35)
	18 or more	2425 (66.2)	(65.5–67.2)

Body mass index	Underweight (< 18.5)	464 (12.7)	(11.8–13.9)
	Normal (18.5–24.9)	2048 (55.9)	(54.7–57.8)
	Overweight (≥ 25)	1153 (31.5)	(29.4–32.4)

Education level	Illiterate	234 (6.4)	(5.5–7.1)
	Primary	816 (22.3)	(21.5–24.1)
	Secondary	1987 (54.2)	(53.1–56.3)
	Higher	627 (17.1)	(15.1–17.4)

Working status	No	2678 (73.1)	(72.2–75)
	Yes	987 (26.9)	(25–27.8)

Access to media	No	1601 (43.7)	(43.3–47)
	Yes	2063 (56.3)	(53 – 56.5)

*Father’s characteristics*			
Education level	Illiterate	580 (15.8)	(14.9–17.2)
	Primary	1161 (31.7)	(31–34)
	Secondary	1277 (34.9)	(33.2–36.2)
	Higher	646 (17.6)	(15.6–18)

Occupation	No‐work	61 (1.7)	(1.2–2)
	Service	1157 (31.6)	(29.9–32.8)
	Non‐agriculture	1711 (46.7)	(45.1–48.3)
	Agriculture	736 (20.1)	(19.1–21.6)

*Household and health-related characteristics*			
Division	Dhaka	900 (24.6)	(22.3–25)
	Chittagong	799 (21.8)	(21.3–25)
	Barisal	249 (6.8)	(6.8–8.5)
	Khulna	360 (9.8)	(8.2–10)
	Mymensingh	314 (8.6)	(7.9–9.7)
	Rajshahi	372 (10.1)	(7.3–11)
	Rangpur	403 (11)	(9.4–11.3)
	Sylhet	268 (7.3)	(8–9.8)

Residence	Urban	958 (26.1)	(21.1–23.7)
	Rural	2706 (73.9)	(73–78.9)

Wealth index	Poor	1511 (41.2)	(41.2–44.3)
	Middle	751 (20.5)	(18.9–21.5)
	Rich	1403 (38.3)	(35.6–38.6)

Religion	Muslim	3375 (92.1)	(91.4–93.1)
	Non‐muslim	289 (7.9)	(6.9–8.6)

Number of household member	≤ 4	1364 (37.2)	(34.8–37.9)
	5–7	1772 (48.3)	(46.8–50)
	8 ≤	528 (14.4)	(14.1–16.4)

Number of child under 5 in household	≤ 2	3481 (95)	(93.7–95.2)
	3 ≤	183 (5)	(4.8–6.3)

Household head’s sex	Male	3208 (87.5)	(85.9–88)
	Female	457 (12.5)	(12–14.1)

Source of drinking water	Improved	3590 (98)	(97.5–98.4)
	Unimproved	74 (2)	(1.6–2.5)

Type of toilet facility	Improved	3038 (82.9)	(81–83.4)
	Unimproved	626 (17.1)	(16.6–19)

Had diarrhea recently	No	3469 (94.7)	(94–95.4)
	Yes	196 (5.3)	(4.6–6)

Had fever in the last 2 weeks	No	2518 (68.7)	(66.9–69.9)
	Yes	1146 (31.3)	(30.1–33.1)

*Note:* Frequencies presented in Table 1 are weighted and rounded to the nearest whole number. Therefore, category totals may differ slightly (±1–2) from the overall weighted sample size of 3664 due to rounding.

Table 2 shows that the mean HAZ of the children was −1.159 (SD = 1.247), indicating that, on average, children were below the WHO reference population in terms of linear growth. The HAZs ranged from −5.68 to 5.27. The distribution was slightly positively skewed (skewness = 0.262). The 5.47th and 23.5th percentiles corresponded to HAZ values of −3 and −2, respectively, while the median HAZ was −1.2. These findings indicate that a substantial proportion of children experienced growth deficits relative to the WHO growth standards. The box plot (Figure 2) also indicates that the HAZ is positively skewed and includes outliers. The Kolmogorov–Smirnov test (test statistic = 0.432, *p*‐value < 0.001) confirms that the data do not follow a normal distribution. Additionally, the Breusch–Pagan test is used to assess the assumption of constant variance, and the results indicate that this assumption is violated (BP = 59.842, df = 43, *p*‐value = 0.045). As a result, nonparametric tests are more appropriate for analyzing the factors associated with childhood stunting. To examine the bivariate relationship between HAZs and the independent variables, the Kruskal–Wallis test is applied. Finally, the QR model is chosen to examine the associations between independent variables and HAZ across different points of the HAZ distribution.

**TABLE 2:** Summary statistics of HAZ.

Mean	SD	Min	Max	Skewness	Kurtosis	Percentiles				
						5.47	23.5	50	75	95
−1.159	1.247	−5.68	5.27	0.262	1.247	−3	−2	−1.2	−0.4	0.95

**FIGURE 2:**
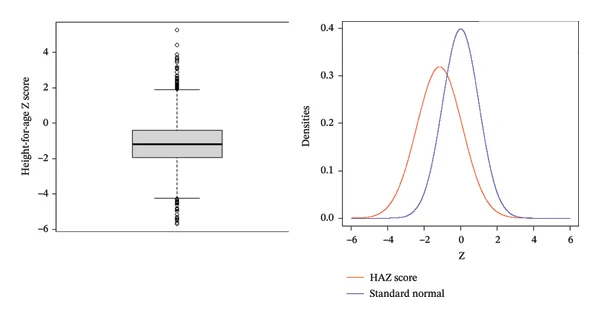
Box plot and density plot of the height‐for‐age Z‐scores with the standard normal variate.

Table 3 presents the comparison of HAZ across child, parental, household, and health‐related characteristics using the Kruskal–Wallis test. The median HAZs vary significantly based on the child’s current age, birth order, twin status, mother’s age at first birth, mother’s BMI, access to media, parental education, father’s occupation, division (region), wealth index, place of residence, and type of toilet facility. Among child characteristics, the median HAZ of children is found to be highest among ≤ 6 months children (−0.703) and lowest among 24–47 months children (−1.330). Higher birth order children (4th+) show substantially lower median HAZ (−1.545) than firstborns (−1.070). Twin children also has notably lower median HAZ (−1.721) compared to single‐born children (−1.200). Regarding maternal characteristics, children born to mothers who have their first birth before 18 years of age have lower median HAZs than those whose mothers have their first birth at age 18 years or later (−1.330 vs. −1.140). Median HAZs improve with increasing maternal BMI and educational attainment. Children of overweight mothers have the highest median HAZ (−0.953), whereas children of underweight mothers have the lowest (−1.530). Similarly, children of mothers with higher education have substantially better HAZs than those of illiterate mothers (−0.740 vs. −1.706). Children whose mothers have access to media also have significantly higher median HAZs than those without media access. Significant differences are also observed according to paternal characteristics. Median HAZs improve with increasing paternal education, ranging from −1.561 among children of illiterate fathers to −0.730 among those whose fathers have higher education. Children whose fathers worked in agriculture have lower median HAZs than those whose fathers are employed in service occupations. Among household characteristics, children residing in urban areas have higher median HAZs than those living in rural areas (−1.024 vs. −1.250). Median HAZs increased with household wealth status, with the poorest households showing the lowest median HAZ (−1.500) and the richest households showing the highest (−0.878). Significant regional variation in HAZs is also observed across divisions. In addition, children from households with improved toilet facilities have significantly higher median HAZs than those from households with unimproved facilities (−1.100 vs. −1.540).

**TABLE 3:** Comparison of height‐for‐age Z‐scores (HAZ) across child, parental, household, and health‐related characteristics using the Kruskal–Wallis test.

Variables		Median HAZ (IQR)	Chi‐square	*p* value
*Child characteristics*				
Age (in months)	≤ 6	−0.703 (−1.618, 0.140)	80.587	< 0.001
	7–23	−1.230 (−1.944, −0.420)		
	24–47	−1.330 (−2.060, −0.550)		
	48–59	−1.162 (−1.890, −0.453)		

Sex	Male	−1.201 (−1.940, −0.360)	0.817	0.365
	Female	−1.199 (−1.910, −0.440)		

Birth order	1st	−1.070 (−1.840, −0.310)	49.194	< 0.001
	2nd‐3rd	−1.190 (−1.929, −0.416)		
	4th or higher	−1.545 (−2.230, −0.808)		

Child is twin	No	−1.200 (−1.921, −0.390)	8.819	0.002
	Yes	−1.721 (−2.320, −0.726)		

*Mother’s characteristics*				
Current age (years)	≤ 18	−1.050 (−1.880, −0.306)	2.405	0.492
	19–24	−1.200 (1.920, −0.390)		
	25–34	−1.199 (−1.910, −0.380)		
	35 or more	−1.357 (−2.040, −0.446)		

Age at first birth	< 18	−1.330 (−2.048, −0.570)	30.564	< 0.001
	18 or more	−1.140 (−1.880, −0.310)		

Body mass index	Underweight (< 18.5)	−1.530 (−2.188, −0.800)	75.157	< 0.001
	Normal (18.5–24.9)	−1.240 (−2.020, −0.450)		
	Overweight (≥ 25)	−0.953 (−1.680, −0.200)		

Education level	Illiterate	−1.706 (−2.509, −0.740)	180.09	< 0.001
	Primary	−1.430 (−2.157, −0.703)		
	Secondary	−1.190 (−1.886, −0.420)		
	Higher	−0.740 (−1.510, −0.010)		

Working status	No	−1.181 (−1.930, −0.370)	1.17	0.279
	Yes	−1.240 (−1.940, −0.450)		

Access to media	No	−1.370 (−2.080, −0.630)	57.713	< 0.001
	Yes	−1.060 (−1.820, −0.260)		

*Father’s characteristics*				
Education level	Illiterate	−1.561 (−2.270, −0.735)	172.97	< 0.001
	Primary	−1.340 (−2.040, −0.590)		
	Secondary	−1.104 (−1.880, −0.360)		
	Higher	−0.730 (−1.580, 0.012)		

Occupation	No‐work	−1.148 (−1.929, −0.362)	100.1	< 0.001
	Service	−0.990 (−1.750, −0.200)		
	Non‐agriculture	−1.260 (−1.970, −0.414)		
	Agriculture	−1.517 (−2.135, −0.770)		

*Household and health-related characteristics*				
Division	Dhaka	−1.000 (‐1.855, −0.170)	33.841	< 0.001
	Chittagong	−1.285 (−1.939, −0.566)		
	Barisal	−1.258 (‐2.015, −0.410)		
	Khulna	−1.148 (−1.833, −0.427)		
	Mymensingh	−1.336 (−2.044, −0.509)		
	Rajshahi	−1.200 (−1.810, −0.324)		
	Rangpur	−1.280 (−1.948, −0.512)		
	Sylhet	−1.405 (−2.253, −0.587)		

Residence	Urban	−1.024 (−1.915, −0.170)	29.427	< 0.001
	Rural	−1.250 (−1.940, −0.503)		

Wealth index	Poor	−1.500 (−2.170, −0.740)	205.97	< 0.001
	Middle	−1.145 (−1.840, −0.550)		
	Rich	−0.878 (−1.680, −0.120)		

Religion	Muslim	−1.200 (−1.911, −0.370)	0.754	0.385
	Non‐muslim	−1.200 (−1.940, −0.390)		

Number of household member	≤ 4	−1.230 (−1.940, −0.390)	0.562	0.754
	5–7	−1.180 (−1.930, −0.360)		
	8 ≤	−1.200 (−1.918, −0.509)		

Number of child under 5 in household	≤ 2	−1.200 (−1.930, −0.380)	0.070	0.791
	3 ≤	−1.090 (−1.942, −0.589)		

Household head’s sex	Male	−1.200 (−1.950, −0.380)	1.374	0.241
	Female	−1.150 (−1.830, −0.432)		

Source of drinking water	Improved	−1.190 (−1.920, −0.390)	1.373	0.241
	Unimproved	−1.719 (−2.037, −0.585)		

Type of toilet facility	Improved	−1.100 (−1.860, −0.330)	78.868	< 0.001
	Unimproved	−1.540 (−2.270, −0.780)		

Had diarrhea recently	No	−1.177 (−1.814, −0.297)	0.727	0.3936
	Yes	−1.200 (−1.940, −0.410)		

Had fever in the last 2 weeks	No	−1.220 (−1.910, −0.450)	0.034	0.853
	Yes	−1.190 (−1.940, −0.360)		

Table 4 shows the equality‐of‐slopes tests, which indicate significant differences in regression coefficients across most quantiles of the HAZ distribution. In particular, significant differences are observed among the lower quantiles (Q_5.47_), the median and upper quantiles (Q_50_, Q_75_, and Q_95_) (all *p* < 0.001). Similarly, significant differences were found between Q_23.5_ and the higher quantiles, as well as between Q_50_, Q_75_, and Q_95_. These findings suggest that the associations between independent variables and HAZs vary across the HAZ distribution, supporting the use of QR rather than a single mean‐based regression model. The only nonsignificant comparison is between Q_23.5_ and Q_50_ (*p* = 0.165), indicating relatively similar associations at these two points of the HAZ distribution.

**TABLE 4:** Test of equality of slope at multiple quantile regression models.

Sl. no.	Hypothesis	*F* (43, 7251)	*p* value
a.	Q_5.47_ = Q_23.5_	1.627	0.006
b.	Q_5.47_ = Q_50_	2.005	< 0.001
c.	Q_5.47_ = Q_75_	3.207	< 0.001
d.	Q_5.47_ = Q_95_	5.214	< 0.001
e.	Q_23.5_ = Q_50_	1.208	0.165
f.	Q_23.5_ = Q_75_	1.756	0.001
g.	Q_23.5_ = Q_95_	4.50	< 0.001
h.	Q_50_ = Q_75_	2.284	< 0.001
i.	Q_50_ = Q_95_	4.31	< 0.001
j.	Q_75_ = Q_95_	3.574	< 0.001

Table 5 presents the results of the multiple QR model, which indicates that the effects of independent variables vary across different points of the conditional distribution of the HAZ. The results show a highly significant association between the child’s age and the HAZ across all quantiles, with children aged 7–59 months have lower HAZ s than those aged 6 months or younger. Being a female child has a significant negative effect of −0.257 at the 95th quantile, indicating that female children have lower HAZs compared to male children at this quantile. Additionally, there is a significant inverse relationship between HAZs and birth order for children in the 4th or higher birth order across all quantiles, suggesting that an increased birth order is associated with lower HAZs. The results also reveal that twin or multiple births have significant negative effects on the conditional distribution of the HAZ, with coefficients of −0.488 at the 23.5th quantile and −0.632 at the 50th quantile.

**TABLE 5:** Quantile regression estimates for the HAZ of under‐5 Bangladeshi children.

Variables		Quantile regression				
		Q_0.0547_ coefficient (se)	Q_0.235_ coefficient (se)	Q_0.5_ coefficient (se)	Q_0.75_ coefficient (se)	Q_0.95_ coefficient (se)
Intercept		−4.427^∗∗∗^ (0.684)	−1.969^∗∗∗^ (0.320)	−1.398^∗∗∗^ (0.296)	−0.280 (0.320)	−0.055 (0.343)

*Child characteristics*						
Age (in months)	≤ 6 (Ref)					
	7–23	−0.467^∗∗∗^ (0.079)	−0.404^∗∗∗^ (0.100)	−0.479^∗∗∗^ (0.104)	−0.538^∗∗∗^ (0.066)	−0.559^∗∗∗^ (0.084)
	24–47	−0.554^∗∗∗^ (0.068)	−0.50^2^ ^∗∗∗^ (0.094)	−0.548^∗∗∗^ (0.101)	−0.692^∗∗∗^ (0.062)	−0.666^∗∗∗^ (0.085)
	48–59	−0.386^∗∗∗^ (0.082)	−0.401^∗∗∗^ (0.105)	−0.426^∗∗∗^ (0.108)	−0.678^∗∗∗^ (0.073)	−0.584^∗∗∗^ (0.090)

Sex	Male (Ref)					
	Female	0.013 (0.052)	0.005 (0.043)	−0.064 (0.037)	−0.058 (0.039)	−0.257^∗∗∗^ (0.055)

Birth order	1st (Ref)					
	2nd‐3rd	−0.030 (0.069)	−0.057 (0.068)	−0.090 (0.055)	−0.038 (0.055)	0.011 (0.082)
	4th or higher	−0.216^∗^ (0.112)	−0.204^∗^ (0.100)	−0.449^∗∗∗^ (0.084)	−0.262^∗^ (0.120)	0.267^∗∗∗^ (0.102)

Twin	No (Ref)					
	Yes	−0.734 (1.185)	−0.488^∗∗^ (0.207)	−0.632^∗∗∗^ (0.106)	−0.394 (0.296)	−0.349 (0.731)

*Mother’s characteristics*						
Current age (years)	≤ 18 (Ref)					
	19–24	−0.238 (0.441)	−0.280 (0.148)	−0.017 (0.100)	−0.037 (0.196)	−0.045 (0.096)
	25–34	−0.179 (0.448)	−0.193 (0.160)	0.088 (0.114)	−0.047 (0.204)	−0.045 (0.126)
	35 or more	−0.202 (0.459)	−0.157 (0.177)	0.211 (0.129)	0.027 (0.213)	0.408^∗∗∗^ (0.137)

Age at first birth	< 18 (Ref)					
	18 or more	0.097 (0.064)	0.042 (0.058)	0.009 (0.048)	0.029 (0.050)	0.236^∗∗∗^ (0.062)

BMI	Underweight (< 18.5) (Ref)					
	Normal (18.5–24.9)	0.107 (0.103)	0.110 (0.066)	0.132^∗^ (0.059)	0.196^∗∗∗^ (0.076)	0.215 (0.151)
	Overweight(≥ 25)	0.423^∗∗∗^ (0.110)	0.323^∗∗∗^ (0.073)	0.289^∗∗∗^ (0.065)	0.266^∗∗∗^ (0.079)	0.320^∗^ (0.165)

Education level	Illiterate (Ref)					
	Primary	0.406^∗∗∗^ (0.147)	0.273^∗∗^ (0.090)	0.142 (0.093)	−0.096 (0.083)	0.222^∗^ (0.101)
	Secondary	0.523^∗∗∗^ (0.147)	0.370^∗∗∗^ (0.092)	0.251^∗∗^ (0.095)	0.050 (0.083)	0.289^∗∗∗^ (0.099)
	Higher	0.594^∗∗∗^ (0.168)	0.637^∗∗∗^ (0.118)	0.416^∗∗∗^ (0.106)	0.175 (0.111)	0.220 (0.119)

Currently working	No (ref)					
	Yes	0.095 (0.056)	0.079 (0.053)	0.062 (0.039)	0.028 (0.058)	−0.019 (0.077)

Access to media	No (ref)					
	Yes	0.147^∗∗^ (0.049)	0.054 (0.048)	0.032 (0.042)	0.139^∗∗^ (0.043)	0.132^∗^ (0.059)

*Father’s characteristics*						
Education level	Illiterate (Ref)					
	Primary	0.199^∗^ (0.097)	−0.018 (0.063)	−0.020 (0.060)	0.037 (0.050)	0.054 (0.084)
	Secondary	0.133 (0.094)	0.007 (0.074)	0.047 (0.060)	0.064 (0.057)	0.168 (0.095)
	Higher	0.238^∗^ (0.101)	0.009 (0.100)	0.141 (0.080)	0.220^∗^ (0.094)	0.726^∗∗∗^ (0.134)

Occupation	No‐work					
	Service	0.393^∗∗∗^ (0.111)	−0.157 (0.235)	−0.108 (0.145)	0.085 (0.098)	0.760^∗∗∗^ (0.109)
	Non‐agriculture	0.110 (0.108)	−0.276 (0.235)	−0.183 (0.145)	0.071 (0.092)	0.663^∗∗∗^ (0.106)
	Agriculture	0.183 (0.138)	−0.364 (0.238)	−0.275 (0.148)	−0.037 (0.103)	0.649^∗∗∗^ (0.156)

*Household and health-related characteristics*						
Division	Dhaka (Ref)					
	Barisal	0.020 (0.074)	−0.002 (0.107)	−0.131 (0.090)	−0.081 (0.091)	−0.000 (0.133)
	Chittagong	0.082 (0.078)	−0.028 (0.102)	−0.195^∗^ (0.079)	−0.180^∗∗^ (0.057)	−0.392^∗∗^ (0.121)
	Khulna	−0.162^∗^ (0.075)	0.020 (0.115)	−0.173^∗^ (0.085)	−0.261^∗∗∗^ (0.077)	−0.071 (0.201)
	Mymensingh	−0.096 (0.169)	−0.007 (0.126)	−0.177^∗^ (0.089)	−0.130 (0.067)	−0.346^∗^ (0.136)
	Rajshahi	0.123 (0.177)	0.112 (0.111)	−0.165^∗^ (0.078)	−0.156 (0.133)	−0.189 (0.140)
	Rangpur	0.166 (0.145)	0.070 (0.109)	−0.189^∗^ (0.086)	−0.052 (0.087)	−0.053 (0.127)
	Sylhet	−0.139 (0.075)	−0.123 (0.113)	−0.256^∗∗^ (0.090)	−0.162^∗^ (0.073)	−0.071 (0.143)

Residence	Urban (Ref)					
	Rural	0.066 (0.059)	0.074 (0.055)	0.064 (0.046)	0.000 (0.049)	−0.167^∗^ (0.065)

Wealth index	Poor					
	Middle	0.056 (0.066)	0.001 (0.072)	0.135^∗∗^ (0.049)	0.125^∗^ (0.057)	−0.008 (0.066)
	Rich	0.257^∗∗∗^ (0.064)	0.235^∗∗∗^ (0.062)	0.284^∗∗∗^ (0.055)	0.395^∗∗∗^ (0.059)	0.247^∗∗^ (0.083)

Religion	Non‐Muslim					
	Muslim	0.433^∗^ (0.217)	−0.063 (0.090)	−0.027 (0.060)	−0.152 (0.102)	−0.152 (0.092)

Number of household member	≤ 4					
	5–7	−0.027 (0.060)	−0.006 (0.048)	−0.012 (0.045)	−0.010 (0.049)	0.030 (0.067)
	8 ≤	0.099 (0.080)	0.040 (0.080)	0.034 (0.071)	−0.085 (0.055)	−0.053 (0.094)

Number of child under 5 in household	≤ 2					
	3 ≤	0.131 (0.091)	0.016 (0.092)	0.082 (0.081)	−0.079 (0.071)	−0.255^∗^ (0.119)

Household head’s sex	Male (Ref)					
	Female	−0.079 (0.062)	0.040 (0.055)	0.031 (0.047)	0.002 (0.090)	−0.014 (0.084)
Source of drinking water	Unimproved					
	Improved	0.259 (0.429)	0.123 (0.089)	0.291 (0.194)	0.032 (0.195)	0.233 (0.211)

Type of toilet facility	Unimproved					
	Improved	0.134 (0.1243)	0.111 (0.058)	0.129^∗^ (0.056)	0.119 (0.068)	−0.016 (0.080)

Had diarrhea recently	No (Ref)					
	Yes	0.207 (0.091)	0.074 (0.076)	0.072 (0.095)	0.166 (0.118)	0.123 (0.186)

Had fever in the last 2 weeks	No (Ref)					
	Yes	0.051 (0.052)	0.031 (0.048)	0.038 (0.038)	0.011 (0.044)	0.068 (0.060)

*Note:* Ref.: reference category.

^∗^refers *p*‐value < 0.05.

^∗∗^refers *p*‐value < 0.01.

^∗∗∗^refers *p*‐value < 0.001.

Mother’s current age of 35 or older is significantly associated with higher HAZs at the 95th quantile, suggesting that older mothers are linked to better child nutritional outcomes. Compared to children of underweight mothers, those with normal BMI mothers exhibit significant increases in Z‐scores of 0.132 and 0.196 at the 50th and 75th quantiles, respectively. Moreover, mothers with a BMI of 25 kg/m^2^ or higher exhibit significant positive coefficients through all quantiles. Access to media for mothers positively influences their children’s HAZs, particularly at the lower (5.47th) and higher (75th and 95th) quantiles. Additionally, children of educated mothers demonstrate significant positive coefficients in most quantiles, with coefficients ranging from 0.238 at the 10th quantile to 0.726 at the 95th quantile for children of highly educated fathers compared to those with illiterate fathers. This indicates that higher parental education levels correlate with higher HAZs rates in children. At the 95th quantile, children with employed fathers also show significant positive coefficients compared to those with unemployed fathers which is suggesting that children of employed fathers have higher HAZs.

Children who live outside of the Dhaka division show significant negative coefficients at various quantiles that indicate lower HAZs compared to those from the Dhaka division. Furthermore, children residing in rural areas are significantly shorter than those in urban areas, but this is only observed at the 95th quantile. Children from wealthy families have significant positive coefficients across all quantiles compared to those from poorer families, which is expected, as higher income typically allows for greater expenditure on nutritious foods. Additionally, households with three or more children under five have HAZs that are significantly lower by 0.255 points compared to those with two or fewer children. It is also noted that children from Muslim households have significantly higher HAZs, but this is observed only at the 5.4th quantile. At the 50th quantile, children from households with improved toilet facilities exhibit positive coefficients, indicating higher HAZs compared to those from households with unimproved toilet facilities.

Figure 3 illustrates the plot of several significant covariates across the 5.47th, 23.5th, 50th, 75th, and 95th QR models (represented by black dots) along with their 95% confidence intervals (indicated by the gray shaded regions). The solid red lines represent the ordinary least squares regression lines, while the associated 95% confidence intervals are shown as dashed red lines. This plot demonstrates that the coefficients vary across the different quantiles.

FIGURE 3Coefficient plot of significant covariates obtained from quantile regression models.

**Figure:**
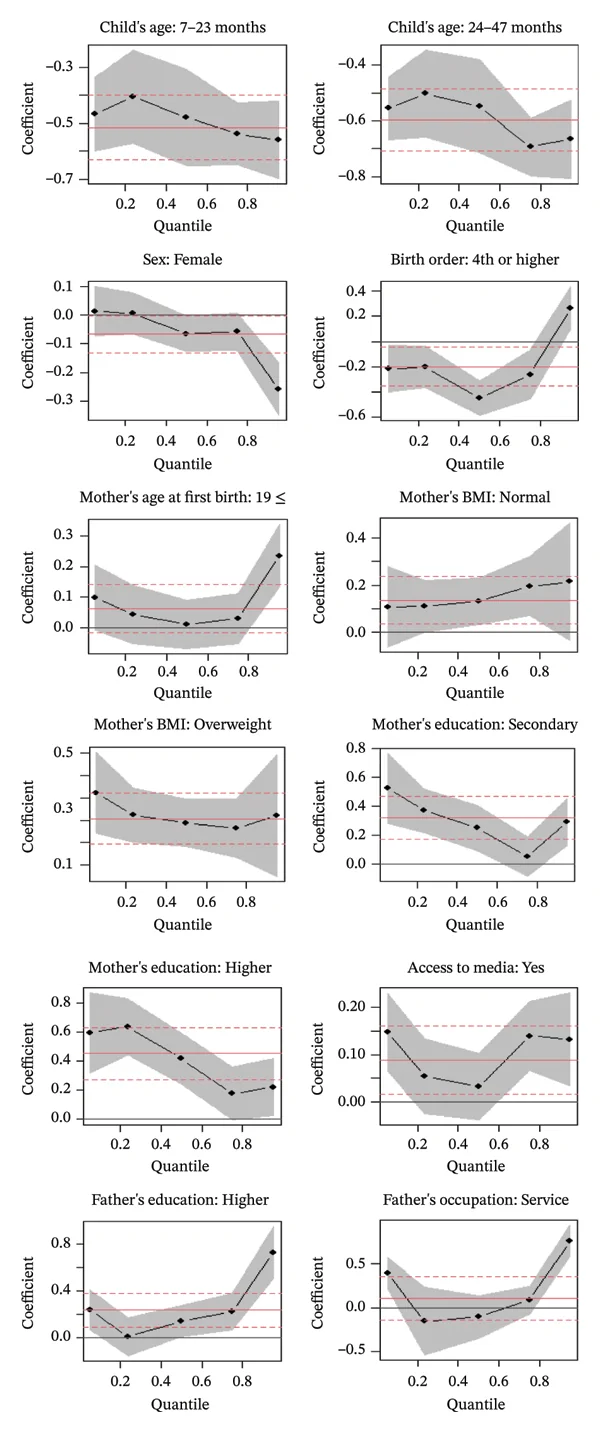

**Figure:**
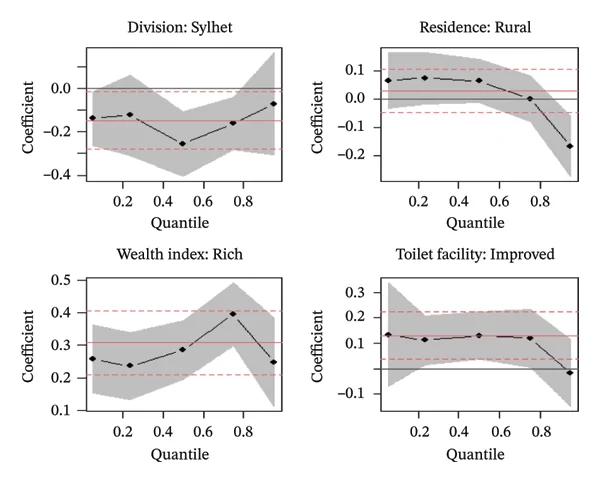

## 4. Discussion

Stunting is impaired growth and development in children that can have long‐term consequences. According to a socioeconomic status assessment conducted in 2016, one in three children in Bangladesh is stunted [28]. This study indicates that the prevalence rate of stunting among Bangladeshi children under 5 years old is 23%, which exceeds the WHO’s threshold of 15% for stunting [19]. The objective of this study is to examine the impact of different independent variables on stunting among under‐five children in Bangladesh by utilizing QR models.

The findings highlight that children aged 7–59 months have lower HAZ compared to those aged 6 months or younger across all quantiles which is consistent with previous studies conducted in India and Egypt [6, 29]. This lower HAZ is likely due to inadequate nutrition, as breastfeeding alone becomes insufficient after 6 months. According to Wit et al. [4], HAZ may increase during the first 6 months of life and subsequently decline during early childhood, potentially reflecting the combined influence of inadequate nutrition, recurrent infections, and adverse environmental conditions. Roth et al. [30] demonstrated that declines in HAZ during early childhood may occur across the entire population distribution rather than within a specific subgroup of children, suggesting population‐wide growth faltering processes. Furthermore, age‐related changes in HAZ may partly reflect growth deceleration toward genetically determined target height, as reported in recent studies [31]. Additionally, the results reveal that female children exhibit significantly lower HAZs than male children at the 95th quantiles of the conditional HAZ distribution, aligning with findings from previous studies in Bangladesh and Sri Lanka [26, 32]. Gender discrimination in households, particularly concerning food allocation, may contribute to this disparity. Furthermore, twin or multiple births are strongly associated with lower HAZ at the 23.5th and 50th quantiles, indicating competition for food among siblings. Research in sub‐Saharan Africa shows that children from multiple births are 2.09 times more likely to be stunted compared to those from single births [33]. The current study also finds that children with higher birth orders has lower HAZ through all quantiles compared to first‐born children which is supported by earlier research in Bangladesh [26]. This may be attributed to the reduced care these children receive.

Moreover, the findings indicate that mother’s current age (35 or older) and age at first birth (19 or older) positively influence in the 95th quantile of the conditional HAZ distribution. Previous studies have shown that delaying the age of first birth by 1 year can reduce stunting rates by 7% in Kenya [34] and 9% in Ethiopia [35]. Additionally, children from affluent households have higher HAZs across all quantiles compared to those from poorer households, likely due to better access to nutritious food as well as improved living conditions. The study also reveals significant positive relationship between a mother’s BMI and parental education with HAZ. Previous studies reported that children of mothers with a normal BMI are 16% less likely to be stunted compared to those with underweight mothers, also children whose parents have lower educational levels and belong to poorer wealth indexes face a higher risk of stunting [36, 36, 37]. Furthermore, mothers’ access to media positively affects their children’s HAZs at most quantiles, which corroborate findings from earlier study in Bangladesh [26], as media can provide valuable information about child health. At the 95th quantile, children of fathers with a professional occupation show significant positive coefficients compared to those with unemployed fathers. This finding supports evidence reported in an earlier Bangladeshi study [36].

The study also highlights regional disparities. Results show that children living in the Dhaka division have significantly higher HAZs compared to those in other divisions, which aligns with earlier research [20]. Additionally, rural children exhibit lower HAZs in the 95th quantile compared to urban children. Previous studies in Vietnam [39] and Bangladesh [40] have reported that rural children are more malnourished than their urban counterparts, potentially due to income and educational disparities. Interestingly, the study finds that Muslim children have significantly higher HAZs than their counterparts at the 5.4th quantile; however, this contrasts with many other studies in Bangladesh [11, 26].

### 4.1. Strengths

This study is based on Bangladesh’s most recent nationally representative surveys and accommodated comprehensive set of variables covering child, parental, household, and health characteristics to provide a comprehensive view of the underlying causes of underweight within children under the age five. To strengthen the authenticity of the findings, both bivariate and multivariate techniques are utilized. In this study, the QR model is chosen because it provides unbiased estimates even when the data do not meet the normality assumption and gives robust conditional estimates in the presence of outliers. In addition, the dataset was weighted according to DHS protocols, ensuring precise and nationally representative results.

### 4.2. Limitations

As this study uses cross‐sectional data, it cannot determine cause‐and‐effect relationships between the risk factors and stunting. Major limitation of this study is the presence of missing values, particularly in the anthropometric data for children which reduces the dataset’s size. Also, important variables such as birth size, dietary diversity, breastfeeding duration, and birth intervals could not be included due to missing data.

## 5. Conclusion

This study found that child, parental, household, and health‐related characteristics were significantly associated with HAZs among children under 5 years of age in Bangladesh. The QR analysis revealed that the magnitude of these associations varied across the HAZ distribution, with several factors showing stronger associations at the lower quantiles, representing children with poorer growth outcomes. These findings demonstrate the value of QR in identifying heterogeneous patterns that may not be detected by conventional mean‐based approaches. Targeted strategies focusing on children with lower HAZs may be particularly important for reducing growth deficits and improving child nutritional status in Bangladesh. The authors believe that the findings of this study will support policymakers in achieving Sustainable Development Goal 3, which is focused on improving health outcomes for children in Bangladesh by 2030.

## Author Contributions

Israt Jahan: conceptualization, data curation, formal analysis, investigation, validation, visualization, writing–original draft, and writing–review and editing. Md. Israt Rayhan: funding acquisition, investigation, and writing–original draft. Mirza Nazmul Hasan: methodology and writing–review and editing. Kanis Fatama Ferdushi: validation and writing–review and editing. Nahid Sultana: supervision and writing–review and editing.

## Funding

This research received fund from North South University (NSU) Conference Travel and Research Grants (CTRG).

## Ethics Statement

This study utilized secondary data from a cross‐sectional, nationally representative dataset (BDHS) that is publicly accessible and anonymized, ensuring no identifiable personal information. As such, ethical approval was not required for this analysis.

## Consent

The authors have nothing to report.

## Conflicts of Interest

The authors declare no conflicts of interest.

## Data Availability

The data that support the findings of this study are openly available in DHS at https://dhsprogram.com/data/available-datasets.cfm.

## References

[1] World Health Organization , Malnutrition, 2021, WHO, https://www.who.int/news-room/fact-sheets/detail/malnutrition.

[2] Sarma H. , Khan J. R. , Asaduzzaman M. et al., Factors Influencing the Prevalence of Stunting Among Children Aged Below Five Years in Bangladesh, Food and Nutrition Bulletin. (2017) 38, no. 3, 291–301, 10.1177/0379572117710103. 28758423

[3] Batiro B. , Demissie T. , Halala Y. , and Anjulo A. A. , Determinants of Stunting Among Children Aged 6–59 Months at Kindo Didaye Woreda, Wolaita Zone, Southern Ethiopia: Unmatched Case Control Study, PLoS One. (2017) 12, no. 12, 1–15, 10.1371/journal.pone.0189106. PMC5737969 29261680

[4] Wit J. M. , Himes J. H. , Buuren S. V. , Denno D. M. , and Suchdev P. S. , Practical Application of Linear Growth Measurements in Clinical Research in Low- and Middle-Income Countries, Hormone Research in Paediatrícs. (2017) 88, no. 1, 79–90, 10.1159/000456007. PMC5804842 28196362

[5] World Health Organization , WHO Child Growth Standards: Length/Height-for-Age, Weight-for-Age, Weight-for-Length, Weight-for-Height and Body Mass Index-for-Age: Methods and Development, 2006, WHO, 10.1111/j.1651-2227.2006.tb02378.x.

[6] Fenske N. , Burns J. , Hothorn T. , and Rehfuess E. A. , Understanding Child Stunting in India: A Comprehensive Analysis of Socio-Economic, Nutritional and Environmental Determinants Using Additive Quantile Regression, PLoS One. (2013) 8, no. 11, 1–16, 10.1371/journal.pone.0078692. PMC3817074 24223839

[7] Leroy J. L. and Frongillo E. A. , Perspective: What Does Stunting Really Mean? A Critical Review of the Evidence, Advances in Nutrition. (2019) 10, no. 2, 196–204, 10.1093/advances/nmy101. PMC6416038 30801614

[8] Millward D. J. , Nutrition, Infection and Stunting: the Roles of Deficiencies of Individual Nutrients and Foods, and of Inflammation, as Determinants of Reduced Linear Growth of Children, Nutrition Research Reviews. (2017) 30, no. 1, 50–72, 10.1017/S0954422416000238. 28112064

[9] Raiten D. J. and Bremer A. A. , Exploring the Nutritional Ecology of Stunting: New Approaches to an Old Problem, Nutrients. (2020) 12, no. 2, 10.3390/nu12020371. PMC7071191 32023835

[10] Islam A. and Biswas T. , Chronic Stunting Among Under-5 Children in Bangladesh: A Situation Analysis, Advances in Pediatric Research. (2015) 2, no. 18, 1–8, https://www.researchgate.net/publication/281321021.

[11] Kumar P. , Rashmi R. , Muhammad T. , and Srivastava S. , Factors Contributing to the Reduction in Childhood Stunting in Bangladesh: A Pooled Data Analysis From the Bangladesh Demographic and Health Surveys of 2004 and 2017-18, BMC Public Health. (2021) 21, no. 1, 1–14, 10.1186/s12889-021-12178-6. PMC8594170 34784935

[12] Alam M. A. , Richard S. A. , Fahim S. M. et al., Impact of Early-Onset Persistent Stunting on Cognitive Development at 5 Years of Age: Results From a Multi-Country Cohort Study, PLoS One. (2020) 15, no. 1, 1–16, 10.1371/journal.pone.0227839. PMC6980491 31978156

[13] Nguyen P. H. , Scott S. , Headey D. et al., The Double Burden of Malnutrition in India: Trends and Inequalities (2006–2016), PLoS One. (2021) 16, no. 2, 1–14, 10.1371/journal.pone.0247856. PMC7906302 33630964

[14] Phalkey R. K. , Aranda-Jan C. , Marx S. , Höfle B. , and Sauerborn R. , Systematic Review of Current Efforts to Quantify the Impacts of Climate Change on Undernutrition, Proceedings of the National Academy of Sciences. (2015) 112, no. 33, 4522–4529, 10.1073/pnas.1409769112. PMC4547305 26216952

[15] United Nations Children’s Fund (UNICEF) , World Health Organization (WHO) , and The World Bank , Levels and Trends in Child Malnutrition: Key Findings of the 2021 Edition of the Joint Child Malnutrition Estimates, 2021, WHO, https://www.who.int/publications/i/item/9789240025257.

[16] National Institute of Population Research and Training (NIPORT) and ICF , Bangladesh Demographic and Health Survey 2022. Dhaka(Bangladesh): NIPORT and ICF, 2023, https://dhsprogram.com/pubs/pdf/PR148/PR148.pdf.

[17] Das S. , Baffour B. , and Richardson A. , Prevalence of Child Undernutrition Measures and Their Spatio-Demographic Inequalities in Bangladesh: An Application of Multilevel Bayesian Modelling, BMC Public Health. (2022) 22, no. 1, 1–21, 10.1186/s12889-022-13170-4. PMC9118603 35585516

[18] Guha S. , Das S. , Baffour B. , and Chandra H. , Multivariate Small Area Modelling of Undernutrition Prevalence Among Under-Five Children in Bangladesh, International Journal of Biostatistics. (2022) 19, no. 1, 191–215, 10.1515/ijb-2021-0130. 35624076

[19] World Food Programme Bangladesh , World Food Programme Bangladesh Nutrition Strategy 2012-2016, 2012, WFP Bangladesh.

[20] Chowdhury T. R. , Chakrabarty S. , Rakib M. , Winn S. , and Bennie J. , Risk Factors for Child Stunting in Bangladesh: An Analysis Using MICS 2019 Data, Archives of Public Health. (2022) 80, no. 1, 1–12, 10.1186/s13690-022-00870-x. PMC9028076 35449114

[21] Islam T. , Anni T. T. , and Hasan S. A. , Prevalence of and Factors Associated With Stunting in Bangladesh: Evidence From Bangladesh Demographic and Health Survey Data Sets (2007, 2011, 2014, and 2017-18), Journal of Public Health. (2023) 32, no. 10, 1877–1889, 10.1007/s10389-023-01957-7.

[22] Rahman M. A. , Halder H. R. , Rahman M. S. , and Parvez M. , Poverty and Childhood Malnutrition: Evidence-Based on a Nationally Representative Survey of Bangladesh, PLoS One. (2021) 16, no. 8, 1–18, 10.1371/journal.pone.0256235. PMC8382176 34424928

[23] Mansur M. , Afiaz A. , and Hossain M. S. , Sociodemographic Risk Factors of Under-Five Stunting in Bangladesh: Assessing the Role of Interactions Using a Machine Learning Method, PLoS One. (2021) 16, no. 8, 1–17, 10.1371/journal.pone.0256729. PMC8407547 34464402

[24] Hossain M. M. , Variance in the Presence of Outlier: Weighted Variance, Journal of Statistics Applications & Probability Letters. (2017) 4, no. 2, 57–59, 10.18576/jsapl/040203.

[25] Ragland D. R. , Dichotomizing Continuous Outcome Variables: Dependence of the Magnitude of Association and Statistical Power on the Cutpoint, Epidemiology. (1992) 3, no. 5, 434–440, 10.1097/00001648-199209000-00009. 1391136

[26] Abdulla F. , Rahman A. , and Hossain M. M. , Prevalence and Risk Predictors of Childhood Stunting in Bangladesh, PLoS One. (2023) 18, no. 1, 1–20, 10.1371/journal.pone.0279901. PMC9879476 36701381

[27] Rodriguez R. N. and Yao Y. , Five Things You Should Know About Quantile Regression, *Proceedings of the SAS Global Forum 2017 Conference*, 2017, https://support.sas.com/resources/papers/proceedings17/SAS0525-2017.pdf.

[28] Rabbani A. , Khan A. , Yusuf S. , and Adams A. , Trends and Determinants of Inequities in Childhood Stunting in Bangladesh From 1996/7 to 2014, International Journal for Equity in Health. (2016) 15, no. 1, 1–14, 10.1186/s12939-016-0477-7. PMC5112749 27852266

[29] Sharaf M. F. , Mansour E. I. , and Rashad A. S. , Child Nutritional Status in Egypt: A Comprehensive Analysis of Socio-Economic Determinants Using a Quantile Regression Approach, Journal of Biosocial Science. (2018) 51, no. 1, 1–17, 10.1017/S0021932017000633. 29316992

[30] Roth D. E. , Krishna A. , Leung M. , Shi J. , Bassani D. G. , and Barros A. J. D. , Early Childhood Linear Growth Faltering in Low-Income and Middle-Income Countries as a Whole-Population Condition: Analysis of 179 Demographic and Health Surveys From 64 Countries (1993–2015), Lancet Global Health. (2017) 5, no. 12, e1249–e1257, 10.1016/S2214-109X(17)30418-7. PMC5695758 29132614

[31] Moelyo A. G. , Sitaresmi M. N. , and Julia M. , Growth Faltering or Deceleration Toward Target Height: Linear Growth Interpretation Using WHO Growth Standard 2006 for Indonesian Children, PLoS One. (2025) 20, no. 4, 1–10, 10.1371/journal.pone.0290053. PMC11970694 40184403

[32] Aturupane H. , Deolalikar A. B. , and Gunewardena D. , McGillivray M. , Dutta I. , and Lawson D. , Determinants of Child Weight and Height in Srilanka: A Quantile Regression Approach, Health Inequality and Development, 2011, Palgrave Macmillan, London, 48–67, 10.1057/9780230304673_4.

[33] Yaya S. , Oladimeji O. , Odusina E. K. , and Bishwajit G. , Household Structure, Maternal Characteristics and Children’s Stunting in Sub-Saharan Africa: Evidence From 35 Countries, International Health. (2022) 14, no. 4, 381–389, 10.1093/inthealth/ihz105. PMC9248065 31927593

[34] Gewa C. A. and Yandell N. , Undernutrition Among Kenyan Children: Contribution of Child, Maternal and Household Factors, Public Health Nutrition. (2012) 15, no. 6, 1029–1038, 10.1017/S136898001100245X. 22107729

[35] Amaha N. D. and Woldeamanuel B. T. , Maternal Factors Associated With Moderate and Severe Stunting in Ethiopian Children: Analysis of Some Environmental Factors Based on 2016 Demographic Health Survey, Nutrition Journal. (2021) 20, no. 1, 1–20, 10.1186/s12937-021-00677-6. PMC7916293 33639943

[36] Chowdhury M. R. K. , Khan H. T. A. , Mondal M. N. I. , and Kabir R. , Socio-Demographic Risk Factors for Severe Malnutrition in Children Aged Under Five Among Various Birth Cohorts in Bangladesh, Journal of Biosocial Science. (2021) 53, no. 4, 590–605, 10.1017/S0021932020000425. 32787991

[37] Sultana P. , Rahman M. M. , and Akter J. , Correlates of Stunting Among Under-Five Children in Bangladesh: A Multilevel Approach, BMC Nutrition. (2019) 5, no. 1, 1–12, 10.1186/s40795-019-0304-9. PMC7050784 32153954

[38] Rahman M. S. , Chowdhury M. R. K. , Billah B. , Kabir R. , Perera N. K. P. , and Kader M. , The Prevalence and Socio-Demographic Risk Factors of Coexistence of Stunting, Wasting and Underweight Among Children Under Five Years in Bangladesh, BMC Nutrition. (2021) 10.21203/rs.3.rs-234367/v1. PMC9394024 35996184

[39] Hien N. N. and Kam S. , Nutritional Status and the Characteristics Related to Malnutrition in Children Under Five Years of Age in Nghean, Vietnam, Journal of Preventive Medicine and Public Health. (2008) 41, no. 4, 232–240, 10.3961/jpmph.2008.41.4.232. 18664729

[40] Rahman A. and Hossain M. , Quantile Regression Approach to Estimating Prevalence and Determinants of Child Malnutrition, Journal of Public Health. (2020) 30, no. 3, 323–339, 10.1007/s10389-020-01277-0.

